# Survival rate and stability of surface-treated and non-surface-treated orthodontic mini-implants: a randomized clinical trial

**DOI:** 10.1590/2177-6709.28.2.e2321345.oar

**Published:** 2023-06-05

**Authors:** Janani RAVI, Sangeetha DURAISAMY, Krishnaraj RAJARAM, Ravi KANNAN, Edeinton ARUMUGAM

**Affiliations:** 1 SRM Dental College, Department of Orthodontics and Dentofacial Orthopaedics (Ramapuram, Chennai, Tamil Nadu, India).

**Keywords:** Sandblasting and acid etching surface treatment, Orthodontic mini-implant insertion torque, Orthodontic mini-implant removal torque, Secondary stability, Failure rate

## Abstract

**Objectives:**

This clinical trial was conducted to evaluate the stability and failure rate of surface-treated orthodontic mini-implants and determine whether they differ from those of non-surface-treated orthodontic mini-implants.

**Trial Design:**

Randomized clinical trial with a split-mouth study design.

**Setting:**

Department of Orthodontics, SRM Dental College, Chennai.

**Participants:**

Patients who required orthodontic mini-implants for anterior retraction in both arches.

**Methods:**

Self-drilling, tapered, titanium orthodontic mini-implants with and without surface treatment were placed in each patient following a split-mouth design. The maximum insertion and removal torques were measured for each implant using a digital torque driver. The failure rates were calculated for each type of mini-implant.

**Results:**

The mean maximum insertion torque was 17.9 ± 5.6 Ncm for surface-treated mini-implants and 16.4 ± 9.0 Ncm for non-surface-treated mini-implants. The mean maximum removal torque was 8.1 ± 2.9 Ncm for surface-treated mini-implants and 3.3 ± 1.9 Ncm for non-surface-treated mini-implants. Among the failed implants, 71.4% were non-surface-treated mini-implants and 28.6% were surface-treated mini-implants.

**Conclusion:**

The insertion torque and failure rate did not differ significantly between the groups, whereas the removal torque was significantly higher in the surface-treated group. Thus, surface treatment using sandblasting and acid etching may improve the secondary stability of self-drilling orthodontic mini-implants.

**Trial registration:**

The trial was registered in the Clinical Trials Registry, India (ICMR NIMS). Registration number: CTRI/2019/10/021718

## RESUMO

**Objetivos:**

Este ensaio clínico foi conduzido para avaliar a estabilidade e a taxa de falha de mini-implantes ortodônticos com superfície tratada, e determinar se elas diferem das dos mini-implantes ortodônticos sem superfície tratada.

**Desenho do estudo:**

Ensaio clínico randomizado com desenho de boca dividida.

**Instituição:**

Department of Orthodontics, SRM Dental College, Chennai/India.

**Participantes:**

Pacientes que necessitavam de mini-implantes ortodônticos para retração anterior em ambas as arcadas.

**Métodos:**

Mini-implantes ortodônticos autoperfurantes, cônicos, de titânio com ou sem tratamento de superfície, foram colocados em cada paciente, seguindo um desenho de boca dividida. Os torques máximos de inserção e de remoção foram medidos para cada mini-implante, usando um torquímetro digital. As taxas de falha foram calculadas para cada tipo de mini-implante.

**Resultados:**

O valor médio do torque máximo de inserção foi de 17,9 ± 5,6 Ncm para mini-implantes com superfície tratada e 16,4 ± 9,0 Ncm para mini-implantes sem superfície tratada. O valor médio do torque máximo de remoção foi de 8,1 ± 2,9 Ncm para mini-implantes com superfície tratada e 3,3 ± 1,9 Ncm para mini-implantes sem superfície tratada. Entre os implantes que falharam, 71,4% eram mini-implantes sem superfície tratada e 28,6% eram mini-implantes com superfície tratada.

**Conclusão:**

O torque de inserção e a taxa de falha não diferiram significativamente entre os grupos; porém, o torque de remoção foi significativamente maior no grupo com superfície tratada. Assim, o tratamento de superfície com jateamento e condicionamento ácido pode melhorar a estabilidade secundária dos mini-implantes ortodônticos autoperfurantes.

**Registro do estudo:**

Esse estudo foi registrado no Clinical Trials Registry, Índia (ICMR NIMS). Número de registro: CTRI/2019/10/021718

## INTRODUCTION

Orthodontic mini-implants have gained popularity over the past three decades due to their low cost, availability for placement at several intraoral sites, minimal invasiveness, ease of placement, and reduced patient compliance.^1^ ^-^ ^4^ Due to the elimination of biomechanical limitations in preserving anchorage, treatment planning in orthodontics saw a major shift from a mechanics-driven approach towards an objective-driven approach.^5^

The failure rate of mini-implants has been reported to vary from 13.5% to 16.4%.^6^ The clinical stability of orthodontic mini-implants depends on numerous factors such as physical characteristics of the mini-implant (length, diameter, screw design, material, surface topography), placement site, cortical bone thickness and density, patient-related factors (age and growth pattern of the mandible), and the placement technique for the mini-implant.^7^ ^-^ ^12^

Primary stability refers to the ability of mini-implants to resist orthodontic forces during immediate loading. The primary stability of orthodontic mini-implants depends on the mechanical retention of the implant to the bone and is limited by the quality of the bone at the placement site, design and size of the mini-implant, and placement technique.^13^ ^,^ ^14^ Insertion torque is an indirect measure of primary stability, and excessively high or low insertion torque results in low stability.^15^ While primary stability is important for orthodontic loading, mechanical retention alone cannot maintain the clinical stability of the mini-implant due to the nature of rotational and dynamic moments generated by orthodontic forces.^1^ ^,^ ^16^

Secondary stability is based on bone remodeling around the implant and is responsible for the clinical stability of the implant during orthodontic treatment. Osseointegration is the direct structural and functional contact between the bone and implant surface. It can withstand dynamic and rotational forces, resulting in improved secondary stability.^17^ Various methods have been used to measure secondary stability and osseointegration, among which the measurement of removal torque is the most widely used.^18^

Surface treatment of implants with sandblasting or sandblasting followed by acid etching removes contaminants, creates surface roughness, and promotes the assimilation of osteoblasts over the implant surface, which results in better bone-to-implant contact and improved clinical stability.^19^ ^-^ ^21^

However, much of the research work evaluating the stability of surface-treated mini-implants has been conducted in bone blocks or animal models, which emphasizes the need for controlled clinical trials, as bone remodeling rates vary considerably in humans.^22^

In a prospective clinical study, Kim et al.^18^ evaluated the removal torque of cylindrical surface-treated C-implants in humans that require predrilling for placement and were subjected to early loading; they reported that a higher removal torque value was associated with these implants.

Park et al.^15^ conducted a prospective clinical trial to determine whether the success rate and primary stability of mini-implants surface-treated by acid etching differed from those of untreated mini-implants. They concluded that neither the success rate nor the primary stability differed between the acid-etched and untreated mini-implants. Secondary stability was not assessed due to heterogeneity in the site of placement of the mini-implants, and 34.7% of the implants were used as anchors for distalization. They recommended that, to evaluate the associations between secondary stability and surface treatment, only those patients who require *en-masse* retraction of their anterior teeth, where the relationship between the tooth and the mini-implant remains relatively unchanged during the treatment, should be included in the study.^15^

Thus, the aim of the present prospective clinical trial was to evaluate the stability and failure rate of surface-treated orthodontic mini-implants and to determine whether they differed from those of non-surface-treated orthodontic mini-implants.

### SPECIFIC OBJECTIVES AND HYPOTHESES

The null hypothesis tested was that the insertion torque, removal torque, and failure rate of surface-treated orthodontic mini-implants would not differ from those of non-surface-treated orthodontic mini-implants.

## MATERIAL AND METHODS

### TRIAL DESIGN

This single-center, split-mouth, randomized clinical trial was conducted at the Department of Orthodontics, SRM Dental College, Ramapuram, Chennai, India. The protocol for the human clinical trial and the methods were approved by the Institutional Review Board and Institutional Ethical Committee, SRM University. The trial was registered in the Clinical Trials Registry, India (ICMR NIMS) with the registration number CTRI/2019/10/021718.

### SAMPLE SIZE CALCULATION

The sample size for the study was determined using the F test and one-way ANOVA, using SPSS software version 5.0. The sample size calculated was 14 per group for an alpha error of 0.01 and a power of 90 for evaluating and comparing the insertion torque, removal torque, and failure rates among the surface-treated and non-surface-treated mini-implants. Considering sample attrition, 18 mini-implants from the study and control group were evaluated.

### PARTICIPANTS, ELIGIBILITY CRITERIA, AND SETTINGS

Patients who required extraction of their maxillary and mandibular first premolars and orthodontic mini-implants for anterior *en-masse* retraction in both arches and who were undergoing fixed orthodontic treatment with a 0.022-in slot MBT prescription were randomly selected for the study. Nine patients who fulfilled the inclusion criteria were recruited, taking sample attrition into consideration. Informed consent for participation in the study was obtained from each patient.

**Figure 1:**
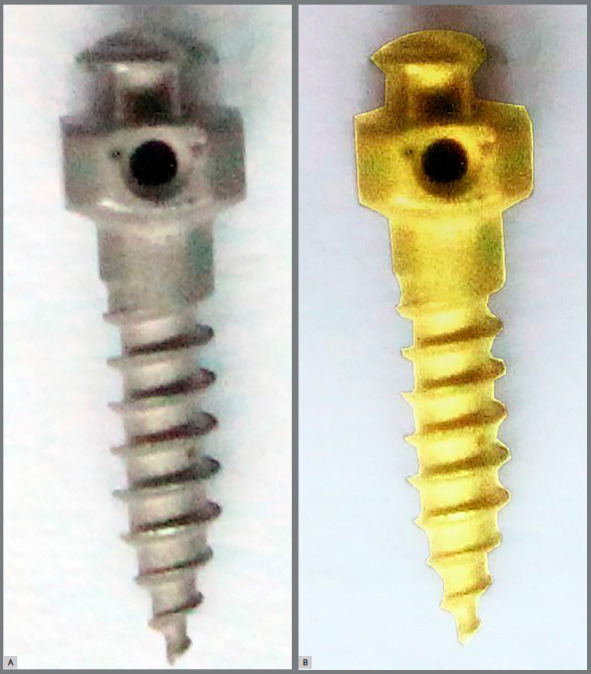
A) Surface treated A1 mini-implant. B) Non-surface treated A1 mini-implant.

The mini-implants used in the study were surface-treated, self-drilling, tapered titanium mini-implants of 2-mm diameter and 8-mm length (A1 orthodontic mini-implants, Bioray Enterprises, Taipei, Taiwan), which were sandblasted with large-grit alumina particles, followed by acid etching with hydrochloric acid and sulfuric acid. This surface treatment was customized by Bioray enterprises for evaluation in the present study. A total number of 18 surface-treated and 18 non-surface-treated mini-implants were placed in these patients following an intra-individual split-mouth design (Fig 1).

### RANDOMIZATION (RANDOM NUMBER GENERATION, ALLOCATION CONCEALMENT, AND IMPLEMENTATION)

**Figure 2:**
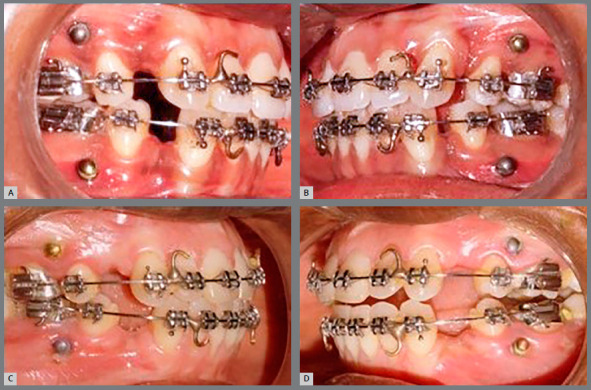
A, B) Type I pattern: surface-treated mini-implants placed in the maxillary right and mandibular left quadrants, and the non-surface-treated mini-implants were placed in the maxillary left and mandibular right quadrants. C, D) Type II pattern: surface-treated mini-implants placed in the maxillary left and mandibular right quadrants, and non-surface-treated mini-implants placed in the maxillary right and mandibular left quadrants.

Patients were randomly assigned to two different types of mini-implant placement patterns. In the type I pattern, the surface-treated mini-implants were placed in the maxillary right and mandibular left quadrants, and the non-surface-treated mini-implants were placed in the maxillary left and mandibular right quadrants (Figs 2A, 2B). In the type II pattern, surface-treated mini-implants were placed in the maxillary left and mandibular right quadrants, and non-surface-treated mini-implants were placed in the maxillary right and mandibular left quadrants (Fig 2C, 2D). Randomization was performed based on the random number table method, and allocation concealment was performed based on the case record numbers of the patients, placed in separate sealed envelopes. 

### INTERVENTION

Orthodontic mini-implants were placed in the interdental region between the second premolar and first molar in all four quadrants by the same orthodontist under local anesthesia following the standard placement protocol.

### OUTCOME

Insertion and removal torques and failure rates were measured for the surface-treated and non-surface-treated mini-implants.

### MEASUREMENT OF INSERTION TORQUE

**Figure 3:**
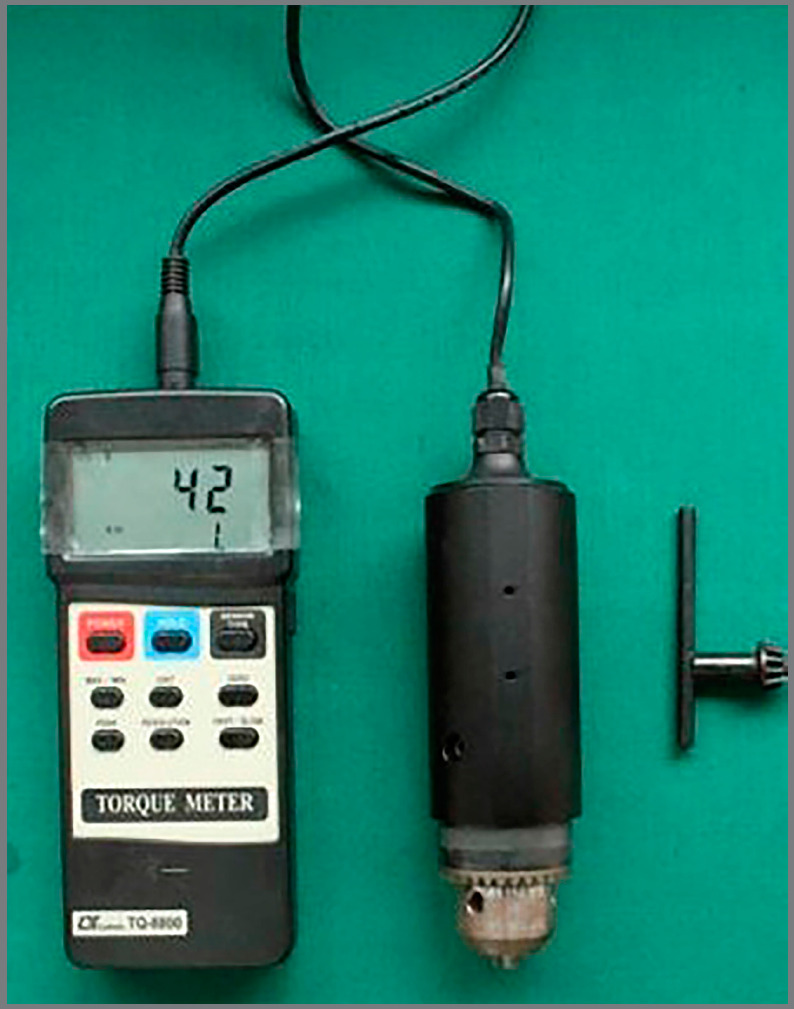
TQ- 8800 Digital torque meter.

**Figure 4:**
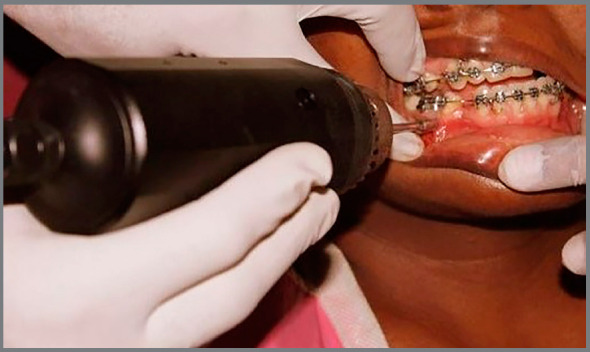
Measurement of insertion torque using Lutron TQ- 8800 digital torque meter.

The mini-implants were loaded onto the detachable long blade tip of the mini-implant drive and attached to the torque probe of a torque meter (Lutron TQ-8800, Lutron Electronic Enterprise Co. Ltd., Taipei, Taiwan; Fig 3). The maximum insertion torque from the initiation to the completion of insertion of the mini-implants was recorded in Ncm (Fig 4). Loading of the orthodontic mini-implants was performed after four weeks of healing period.^23^ Retraction was performed in 0.019 × 0.025-in stainless steel archwire with soldered brass hooks using NiTi closed-coil springs. A retraction force of 150 g per side of the arch was calibrated using the Dontrix gauge (American Orthodontics, Sheboygan, Wisconsin, USA).

### MEASUREMENT OF THE FAILURE RATES

Mini-implants that showed minimal mobility but could resist further load and remained in the bone until the end of treatment were considered successful, whereas those that loosened during the treatment and could not resist the orthodontic force loading were considered failures.^24^

### MEASUREMENT OF REMOVAL TORQUE

All mini-implants were removed at the end of space closure. The maximum removal torque value from the initiation to the completion of removal was recorded in Ncm using a torque meter (Lutron TQ -8800, Lutron Electronic Enterprise Co. Ltd., Taipei, Taiwan).

### INTER RIM ANALYSIS AND STOPPING GUIDELINES

Not applicable

### STATISTICAL METHOD

This study followed a split-mouth study design, in which the study and control groups were placed in the same patient, and the baseline and demographic data for age and sex, compared among the patients. All patients included in the study were female, aged 23-29 years. The maximum insertion and removal torques of the surface-treated and non-surface-treated mini-implants were compared using the Mann-Whitney U test. The failure rates of the two groups were statistically analyzed using the chi-square test - *p* < 0.05 was considered significant.

## RESULTS

### PARTICIPANT FLOW

**Figure 5:**
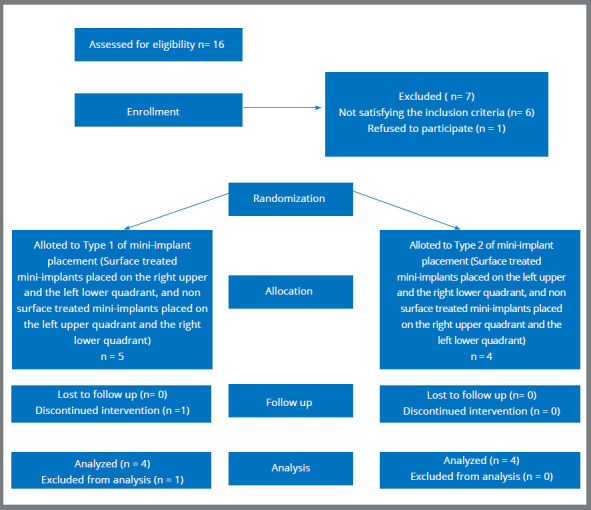
Consort flow diagram.

Out of the 16 patients considered for the study, 9 who satisfied the inclusion criteria were chosen, and 36 orthodontic mini-implants were placed in their mouths. One patient was unable to continue the treatment until completion of space closure and was excluded from the study (Fig 5). A total of 32 mini-implants were thus available for the failure rate analysis. Mini-implants that failed and were relocated to a different site during the course of treatment were excluded from the insertion and removal torque analysis.

### INSERTION AND REMOVAL TORQUE

The mean maximum insertion torque was 17.9 ± 5.6 Ncm for surface-treated mini-implants and 16.4 ± 9.0 Ncm for non-surface-treated mini-implants. The mean maximum removal torque was 8.1 ± 2.9 Ncm for surface-treated mini-implants and 3.3 ± 1.9 Ncm for non-surface-treated mini-implants (Table 1). The maximum insertion torque did not differ significantly between the surface-treated and non-surface-treated orthodontic mini-implants, whereas a statistically significant difference was seen for the maximum removal torque between the surface-treated and non-surface-treated orthodontic mini-implants.

**Table 1:** Bivariate comparison of maximum insertion and removal torque between surface treated and non-surface treated orthodontic mini-implants, using Mann-Whitney U test.

Variables	Groups	n	Mean	SD	Z-value	p-value
Insertion torque	Surface treated	14	17.9	5.6	0.42	0.674 ^#^
	Non surface treated	11	16.4	9.0		
Removal torque	Surface treated	14	8.1	2.9	2.629	0.009 ^**^
	Non surface treated	11	3.3	1.9		

*p*-value < 0.05 considered statistically significant. **Highly significant. *Significant. ^#^not significant.

### FAILURE RATES

Among the failed mini-implants, 71.4% were non-surface-treated mini-implants and 28.6% were surface-treated mini-implants. Although the failure rate was lower for surface-treated mini-implants than for non-surface-treated mini-implants, the difference was not statistically significant (Table 2).

**Table 2:** Comparison of failure rates between surfaces treated and non-surface treated orthodontic mini-implants, using the Chi-Square test.

			Group		Total	Chi-square	p-value	Sig^a^
			*Non-surface treated mini-implants*	*Surface treated orthodontic mini-implants*				
Failure	no failure	Count	11	14	25	1.646	0.394	NS
		% within failure	44.0%	56.0%	100.0%			
	failure	Count	5	2	7			
		% within failure	71.4%	28.6%	100.0%			
Total	Count	16	16	32				
	% within failure	50.0%	50.0%	100.0%				

^a^ Sig indicates significance by the Chi-square test. P < 0.05 indicates statistically significance. NS = not significant.

### HARMS

The only harm that was expected in the trial was accidental root damage during mini-implant placement. No damage to the adjacent roots was found in this trial.

## DISCUSSION

Surface treatment with sandblasting and acid etching allows osteoblast migration and retention over the orthodontic mini-implant surface, facilitating osseointegration, which results in improved clinical stability of the implant.^25^ Different *in vitro* studies in bone blocks and animal studies have shown increased pull-out strength and improved stability of surface-treated orthodontic mini-implants.^26^ ^-^ ^28^ However, clinical studies with stringent inclusion criteria evaluating the secondary stability of surface-treated orthodontic mini-implants are lacking in the literature. This prospective randomized controlled clinical trial was designed to evaluate and compare the insertion and removal torques and failure rates of surface-treated and non-surface-treated self-drilling titanium orthodontic mini-implants placed in the buccal interdental area of the patients’ mouth to obtain anchorage for *en-masse* retraction of the anterior teeth.

Primary stability is defined as the mechanical retention of the implant to the bone. It is an important factor determining the clinical success of an implant and is commonly assessed by measuring the maximum insertion torque. An optimum insertion torque reduces micromotion of the implant in the bone after insertion, which can affect primary mechanical stability. High insertion torque may result in stripping of the bone during insertion, which results in reduced holding strength of the implant and reduces the secondary stability of implants by 40%-50%.^29^

In this study, the mean maximum insertion torque for surface-treated mini-implants and non-surface-treated mini-implants was 17.9 ± 5.6 Ncm and 16.4 ± 9.0 Ncm, respectively (Table 2), with no significant difference, which is similar to the findings of the study published by Park et al.^15^

Although the insertion torque measured in this study was higher than the normally recommended (5-10 Ncm), the failure rate reported was comparable to that reported in previous studies.^6^ ^,^ ^29^ This confirms that the current recommendations on the optimum maximum insertion torque should be reviewed. The survival rates of mini-implants with an insertion torque of >15 Ncm were higher, consistent with previous findings by Chaddad et al.^30^

The removal torque is the rotational force applied for the removal of mini-implants. The maximum removal torque is the highest value of removal torque recorded during implant removal.^28^ A higher removal torque is seen in mini-implants with better secondary stability, and is dependent on numerous factors, including the size of the mini-implant, a good primary stability, and its potential for osseointegration.^12^ ^,^ ^31^

In this study, the mean removal torque of surface-treated and non-surface-treated mini-implants was 8.1 ± 2.9 Ncm and 3.3 ± 1.9 Ncm, respectively (Table 1). The removal torque of surface-treated mini-implants was significantly higher than that of non-surface-treated mini-implants. These results are consistent with those of the study by Kim et al.^18^ and other animal studies evaluating the maximum removal torque and new bone formation around surface-treated implants after insertion.^25^ ^,^ ^31^ ^,^ ^32^

The removal torque is a parameter widely used for evaluating the osseointegration of implants. Osseointegration of orthodontic mini-implants may offer high stability during orthodontic treatment and the ability to withstand dynamic and rotational forces, and may allow more choices for the application of orthodontic force.

The implications of osseointegration in implant removal after the completion of orthodontic treatment are relevant. A very high removal torque may damage the surrounding bone or fracture the mini-implant during removal.^29^ ^,^ ^31^ In the present study, no such difficulties were experienced during the removal of surface-treated mini-implants, although the maximum removal torque of these implants was significantly higher than that of non-surface-treated implants. This may be attributed to the fact that the implants were loaded with orthodontic force immediately after the healing period of four weeks. This may have discouraged complete osseointegration.^23^

In this study, non-surface-treated mini-implants contributed with 71.4% of the failed implants, whereas surface-treated mini-implants contributed with 28.6% of the failed implants. Although the failure rate was lower for surface-treated mini-implants than for non-surface-treated mini-implants, the difference was not statistically significant. This may be attributed to the small sample size of this study (Table 2). The reduced failure rates of surface-treated mini-implants may be due to possible osseointegration and improved bone-to-implant contact.

Extensive research has been conducted in the past, both with dental and orthodontic implants, concluding that increasing the roughness of implants promotes osseointegration.^16^ ^,^ ^17^ ^,^ ^19^ ^-^ ^23^ ^,^ ^31^ ^,^ ^33^ ^,^ ^34^ The present study showed that surface-treated orthodontic mini-implants were associated with an increased removal torque, and no difficulty was encountered during the removal of these mini-implants, suggesting partial osseointegration of the surface-treated mini-implants.

## LIMITATIONS

Further studies with a large sample size are required to strongly associate the surface treatment of implants with the increased secondary stability of orthodontic mini-implants.

## GENERALIZABILITY

This study was conducted at a National Dental Council-accredited dental college. All participants were treated by postgraduate students under the supervision of an experienced faculty member. The patients who participated in the trial may represent a typical orthodontic caseload requiring fixed mechanotherapy and maximum anchorage with orthodontic mini-implants for *en-masse* retraction of the anterior teeth. It can be, therefore, assumed that the results of the present trial are applicable in most clinical settings where mini-implants surface-treated by sandblasting with large-grit alumina and etching with hydrochloric and sulfuric acid can be used in patients requiring maximum anchorage for improved stability.

## CONCLUSION

No significant differences were noted between the insertion torque and failure rates of surface-treated and non-surface-treated orthodontic mini-implants. The removal torque of surface-treated orthodontic mini-implants was significantly higher than that of non-surface-treated implants. Improved secondary stability of orthodontic mini-implants can be achieved with an appropriate surface treatment.
